# Small spherical foreign bodies in the genitourinary tract and their management

**DOI:** 10.1186/s12887-022-03114-7

**Published:** 2022-01-15

**Authors:** You Jia, Li Shuang, Wang Jun, Li Gang, Chen Hai-tao

**Affiliations:** Department of Pediatric Urology, Wuhan Children’s Hospital (Wuhan Maternal and Child Healthcare Hospital), Tongji Medical College, Huazhong University of Science and Technology, No.100, Hongkong Road, Jiang’an District, Wuhan, 430016 Hubei China

**Keywords:** Foreign body, Spherical, Children, Genitourinary, Endoscopy

## Abstract

**Background:**

Urogenital small foreign bodies (FBs) have rarely been reported in children, and their management is still challenging. This study aimed to describe the characteristics and treatment of spherical FBs no larger than 0.6 cm in the children’s genitourinary tracts.

**Methods:**

The clinical data of spherical FBs removed in our hospital from June 2013 to June 2020 were recorded and retrospectively analyzed, including demographics, location, symptoms, imaging examinations and treatment methods.

**Results:**

A total of 10 patients were enrolled: 6 girls and 4 boys. Their ages ranged from 5.1 to 16.8 years old, with a mean age of 9.2 years. The course of the disease ranged from 3 h to 1 year, and symptoms recurred in some cases. Their imaging characteristics were reviewed and analyzed, 6 patients underwent color Doppler ultrasonography, 1 patient was suspected to have an FB in the vagina, 7 patients underwent an X-ray examination, and FBs were revealed in 6 patients. All FBs were removed under endoscopic minimally invasive surgery. Six vaginal FBs were successfully retrieved via vaginoscopy, and in the other four cases, removal by transurethral cystoscopy failed because of mutual attraction, which was eliminated by laparoscopy under pneumovesicum. Postoperative recovery was uneventful; in a follow-up of 3 months to 2 years, there was no perforation or fistula formation, and there were no urethral strictures in boys.

**Conclusion:**

Small spherical FBs are clinically rare; they are sometimes difficult to detect by imaging examinations and can be easily overlooked. Minimally invasive endoscopy remains the first-line approach for the diagnosis and removal of genitourinary spherical FBs.

## Background

Foreign bodies (FBs) in the genitourinary tract of children are rare in the clinical setting. FBs are one of the uncommon causes of increased reproductive discharge and reproductive bleeding in prepubertal girls and are also the reason for complaints of hematuria and frequent urination in boys [1–5]. FBs are prevalent in girls aged at 4–9 years, while in boys, they are mostly prevalent in adolescent children, and they are usually inserted by children themselves or when seeking for sexual gratification; some were caused by sexual abuse, as reported in the literature [5–8]. The presentation differs with the type, composition, size and shape of FBs. It is easier to discover corrosive or sharp FBs, which can damage the mucosal membranes within a few hours, causing severe acute pain and leading to an early diagnosis. Otherwise, if not diagnosed and eliminated in time, FBs can cause serious complications such as repeated genitourinary tract infections, hematuria and dysuria, and even fistula [8–12].

Currently the size of reported FBs is almost over 2 cm, while FBs less than 2 cm especially spherical FBs less than 0.6 cm, in the genitourinary tract are rarely reported [12, 13]. Small spherical FBs are round and blunt with smooth surfaces and do not display any significant clinical symptoms or signs in the early stages. A large clinical study showed that the sensitivity of transabdominal ultrasonography to detect an FB less than 2 cm was 33%, and it is generally difficult to detect FBs that are 0.6 cm or smaller by ultrasound [7] ;therefore, clinicians tend to ignore the presence of FBs that cause the disease to recur or worsen, and their management is still challenging. We treated several patients in our center. We retrospectively analyzed the clinical data to summarize the clinical characteristics and treatment experiences to raise awareness of the disease for early diagnosis and intervention.

## Materials and methods

### Criteria of selection

Data for all patients under 18 years of age who visited our hospital from June 2013 to June 2020, were suspected to have urogenital FBs and were admitted to our hospital for surgical treatment were retrospectively analyzed in this study. Patients with FBs larger than 0.6 cm in size or that were not spherical were excluded from the study.

### Surgical methods

All children intended to undergo cystoscopy or vaginoscopy under general anesthesia, if transurethral cystoscopy removal failed, the laparoscopic technique was performed under pneumovesicum instead. A 10 mm and two 5 mm trocars were required, and the 10 mm port facilitated the removal of FBs via using forceps. All FBs were successfully removed, a final cystoscopy was performed after the removal of the FBs to confirm there were no residual FBs, and then the port site was closed. All operations were uneventful, and there were no surgical complications.

## Results

A total of 354 patients underwent endoscopic examination. A total of 10 patients were included (approximately 2.8%; 6 girls and 4 boys), with an age distribution between 5.1 and 16.8 years old and a mean and median age of 9.2 years and 6.3 years, respectively. Four patients presented repeated reproductive discharge and reproductive bleeding, 3 patients showed symptoms of urinary tract infection, and 3 patients displayed abdominal pain or discomfort. The course of disease ranged from 3 h to 1 year, the median time was 3.5d, and the length of duration was over six months for 4 girls. Six FBs were located in the vagina and 4 were located in the bladder or posterior urethra. The types of FBs were magnetic metallic, nonmagnetic metallic and nonmagnetic plastic beads, with 4 cases, 1 case and 5 cases, respectively. There were unremarkable signs when physical examination was performed. Six patients were treated with antibiotics before consultation, and the treatment was ineffective or effective, while the symptoms tended to relapse when the patients stopped taking the drugs.

Six patients underwent color Doppler ultrasonography, 1 patient had high echo images, suggesting an FB in the vagina, and no abnormalities were found in the rest of the patients; 7 patients underwent an X-ray examination. FBs were found in 6 patients, 2 cases of solitary FBs in the vagina and 4 cases of multiple FBs located in the urinary tract, such as the bladder or urethra. Six intravaginal FBs were successfully retrieved via vaginoscopy: one nonmagnetic metallic ball and five plastic beads. The remaining four FBs were magnetic metallic beads for which removal by transurethral cystoscopy failed because of mutual attraction, and they were retrieved by laparoscopy under pneumovesicum. There was no conversion to open surgery, and no hymen damage; during the operation, the mucosae revealed diverse degrees of congestion and secretion. Postoperative recovery was uneventful; in a follow-up of 3 months to 2 years, there was no perforation or fistula formation, and there were no urethral strictures in boys (Table 1).

**Table 1:** Characteristics of the small spherical foreign bodies in the genitourinary tract and their management

**Case**	**Age** **(Years)**	**Gender**	**Clinical** **presentation**	**Duration**	**Location**	**X-Ray**	**DUS**	**Retrieval** **method**	**Characteristics**	**Number of FBs**
1	12.3	Male	Frequent and painful urination	4 days	Intravesical and urethral	High density shadow	ND	LPV	Magnetic and metallic	38
2	12.4	Male	Abdominal pain	5 h	Intravesical	High density shadow	ND	LPV	Magnetic and metallic	31
3	16.7	Male	Urethral pain and bleeding	3 h	Intravesical and urethral	High density shadow	ND	LPV	Magnetic and metallic	52
4	6.3	Female	Recurrent vaginal discharge and bleeding	8 months	Intravaginal	ND	NF	Vaginoscopy	Nonmagnetic and metallic	1
5	5.2	Female	Abdominal discomfort	24 h	Intravaginal	High density shadow	NF	Vaginoscopy	Nonmagnetic and plastic	1
6	5.7	Female	Abdominal discomfort	4 h	Intravaginal	High density shadow	NF	Vaginoscopy	Nonmagnetic and plastic	1
7	5.9	Female	Recurrent vaginal discharge and bleeding	1 year	Intravaginal	ND	NF	Vaginoscopy	Nonmagnetic and plastic	2
8	5.1	Female	Recurrent vaginal discharge	6 months	Intravaginal	NF	Abnorm-al echoes	Vaginoscopy	Nonmagnetic and plastic	1
9	6.3	Female	Recurrent vaginal discharge and bleeding	1 year	Intravaginal	ND	NF	Vaginoscopy	Nonmagnetic and plastic	1
10	16.8	Male	Recurrent gross hematuria	3 days	Intravesical	High density shadow	ND	LPV	Magnetic and metallic	10

*FB* foreign body, *ND* not done, *NF* not found, *DUS* Doppler ultrasound, *LPV* the laparoscopic approach for intravesical surgery using pneumovesicum

## Discussion

FBs, which lead to approximately 3% of cases of repeated increased reproductive discharge or reproductive bleeding, are clinically rare, accounting for approximately 4% of girls visiting a doctor [1, 3, 4]; Smith et al. [5] reported that approximately 10% of FBs contributed to long-term reproductive discharge in 41 patients; Moreover, Howell et al. [2] in a literature review found that FBs caused reproductive bleeding in prepubertal girls, with a rate as high as 10% in the past 20 years. The clinical manifestations of genitourinary FBs depend on their physicochemical properties and residence time, and there are usually no specific clinical symptoms. FBs can manifest as repeated discharge, hematuria, and recurrent genitourinary infections, dysuria, and so on [2–5, 8–10, 12]. Our cases revealed the same symptoms, the difference is that the FBs were smaller and were easier for physicians to ignore, which made patients suffer more.

Because of the young children's ignorance, sometimes fear or feeling embarrassed, it is rarely possible to acquire a clear history of FB implantation before the operation, which increases the difficulty of diagnosis. The lack of a distinct medical history and a definitive diagnosis leads to reliance on preoperative imaging tests. Color Doppler ultrasonography is considered to be the first choice for the diagnosis of FBs because of its advantages, such as being nonintrusive, not requiring radiation, and being affordable. Yang et al. [7] conducted a retrospective study in which that 249 patients with suspected FBs underwent a color Doppler ultrasound examination to diagnose reproductive FBs, with an overall sensitivity of 81%, which indicated that at least about 20% of FBs will be missed. It is easy to misdiagnose infection in clinical practice; once antibiotic treatment is stopped, symptoms occur repeatedly [9]. In our research, six patients underwent abdominal color Doppler ultrasonography, and only one patient revealed to have a vaginal FB, a result similar to that of the previously reported research [7]. Kyrgios et al. [14] believed that if a reproductive FB is suspected, it is very beneficial to perform a pelvic X-ray scanning before the surgery. The X-ray-of an opaque FB could assist the diagnosis, and verify the shape and size of the FB. The specificity of abnormal discovery detected by plain pelvic radiography was up to 91%; unfortunately, the sensitivity was only 24% [7]. FBs were found in 6 out of 7 patients who underwent X-ray in our study.

For children who underwent color Doppler ultrasound and pelvic X-ray examination, no abnormalities were found, but patients with long-term and repeated unexplained symptoms were recommended to undergo endoscopy as soon as possible. Currently, vaginoscopy is the standard treatment for gynecologic problems and is a safe and effective method for removing female reproductive FBs [15].

Cystoscopy is a widely used endoscope tool in urology with a clear light source and operating channels and is a preferred instrument to be used for confirmed diagnosis and to eliminate various FBs in the genitourinary system [12, 16]. For urogenital magnetic beads, which consist of spherical rare-earth magnets, the smooth surface and mutual attraction made it difficult to remove through the urethra under cystoscopy. After transurethral endoscopic attempts to remove FBs were unsuccessful, Levine et al. [17] believed that transvesical open surgery should be the first-line treatment for the removal of magnetic intravesical FBs. Researchers have also suggested that pneumovesical laparoscopy surgery could be a useful option for special FBs, which may cause urethral injury or fail to be retrieved via the transurethral approach [18, 19].

In our setting, we prefer to use the suprapubic three-port laparoscopic technique under pneumovesicum to remove magnetic FBs so that urethral stricture can be avoided. To establish operating access and inflate the bladder with carbon dioxide, the procedures were the same as those reported in the literature [19]. The FBs were removed once or repeatedly through the 10 mm channel, all operations were successful, and there were no surgical complications or residual FBs during the intraoperative examination. The patients’ postoperative courses were uneventful, and there was no fistula formation or urethral stricture.

We believe that spherical FBs are small and smooth and do not result in obvious acute damage to the mucosae of the urogenital tract in the early stage. Furthermore, patients often do not provide a specific medical history. Therefore, doctors have difficulty in early diagnosis. Once the onset of symptoms occurs, thorough consideration is essential for early confirmation of a diagnosis. If FBs are not diagnosed early, prepubertal girls present repeated reproductive bleeding or increased reproductive secretions and boys manifest unexplained chronic hematuria or frequent urination; conversely, when auxiliary imaging examinations are negative, clinicians should have a high suspicion rate for the possibility of the presence of small FBs in the genitourinary tract. It is essential to perform a surgical exploration as soon as possible. Minimally invasive endoscopy remains the first-line method for the removal of genitourinary FBs.

## Data Availability

The raw dataset analyzed in the current study are available from the corresponding author on reasonable request.
